# Auditory P300 in individuals with high schizotypy: associations of schizotypal traits with amplitude and latency under different oddball conditions

**DOI:** 10.3389/fnhum.2023.1107858

**Published:** 2023-05-18

**Authors:** Jue Deng, Siwei Chen, Yuanhua Ou, Yuanjun Zhang, Ziyue Lin, Yane Shen, Yiduo Ye

**Affiliations:** ^1^Cognitive Neuroscience and Abnormal Psychology Laboratory, Department of Penalty Execution, Fujian Police College, Fuzhou, China; ^2^School of Psychology, Fujian Normal University, Fuzhou, China; ^3^School of Basic Medical Sciences, Fujian Medical University, Fuzhou, China

**Keywords:** schizotypy, auditory P300, duration, frequency, schizophrenia

## Abstract

**Background:**

The aim of this study was to compare the characteristics of auditory P300 between non-clinical individuals with high and low schizotypal traits, and investigate the relationship between schizotypy and P300 under various oddball conditions.

**Methods:**

An extreme-group design was adopted. After screening 1,519 young adults using the Schizotypal Personality Questionnaire (SPQ), sixty-three participants were chosen and divided into two groups (schizotypy group: 31 participants; control group: 32 participants). Basic demographic information was assessed and matched between groups. Depression and anxiety indexes were evaluated and controlled. The P300 component was evoked by an auditory oddball paradigm with different frequencies and durations.

**Results:**

(1) The duration P300 amplitude at PZ site was significantly weaker in the schizotypy group than in the control group [*F*(1,54) = 7.455, *p* = 0.009, η_*p*_^2^ = 0.121]. (2) In the schizotypy group, the latency of frequency P300 at PZ site under large-variant oddball condition was significantly correlated with total SPQ scores (*r*_*p*_ = 0.451, *p* = 0.018) and disorganized dimension scores (*r*_*p*_ = 0.381, *p* = 0.050). (3) In the control group, significantly negative correlations was found between the negative dimension score of SPQ and the frequency P300 amplitudes under small variant condition (PZ: *r*_*p*_ = −0.393, *p* = 0.043; CPZ: *r*_*p*_ = −0.406, *p* = 0.035). In addition, a significant negative relationship was found between disorganized dimension scores and the duration P300 latency at CPZ site under large-variant oddball condition (*r*_*p*_ = −0.518, *p* = 0.006). Moreover, a significant negative association was found between the duration P300 amplitude at CPZ site under small-variant oddball condition and negative factor scores (*r*_*p*_ = −0.410, *p* = 0.034).

**Conclusion:**

Individuals with high schizotypal traits were likely to have deficient attention and hypoactive working memory for processing auditory information, especially the duration of sounds. P300 effects were correlated with negative and disorganized schizotypy, rather than positive schizotypy. There were diverse patterns of relationship between schizotypal traits and P300 under different oddball conditions, suggesting that characteristics and parameters of target stimuli should be considered cautiously when implementing an auditory oddball paradigm for individuals with schizophrenia spectrum.

## 1. Introduction

P300 is a late cognitive component of event-related potentials (ERPs), which is thought to reflect a working memory update of change and attention (Nieman et al., 2002). Deficits in auditory P300 measures are potential neurophysiological markers of schizophrenia (Wang et al., 2003; Higuchi et al., 2013, 2021; Kim et al., 2015). Previous studies reported that lower amplitude and longer latency of P300 in patients with schizophrenia (Bramon et al., 2004; De Wilde et al., 2008; Kim et al., 2015), in their first-degree biological relatives, and in the individuals with clinical high risk for psychosis (Ozgürdal et al., 2008; Hamilton et al., 2019). These studies indicated the schizophrenia-related insufficiency of attention and working memory for acoustic change (Kim et al., 2015), and impaired top-down identification and discrimination of external sounds (Schenkein, 2006; St. George and Cone, 2020).

Schizotypy is a personality organization susceptible to schizophrenia-spectrum disorders (Lenzenweger, 2006; Oestreich et al., 2019; Qin et al., 2022). It has been characterized by schizophrenia-like symptoms in the general population (Ettinger et al., 2018; Polner et al., 2021; Raine et al., 2021). Similar to schizophrenia, schizotypy comprises three dimensions, including positive, negative, and disorganized schizotypy (Raine, 1991; Kwapil and Barrantes-Vidal, 2015; Deng et al., 2022). The positive or cognitive-perceptual dimension is characterized by disruptions in the content of thought, perceptual oddities in all senses (illusions and hallucinations), suspiciousness, and paranoia. The negative or interpersonal dimension is characterized by diminution in experiences including alogia, anergia, avolition, anhedonia, and flattened affect. The disorganization dimension includes disruptions in the ability to organize and express thoughts and behavior, ranging from mild disturbances in thinking and behavior to formal thought disorder and grossly disorganized actions.

Studies on schizotypy-continuum have demonstrated that psychosis exists on a continuum with normal perceptual experiences (Kwapil et al., 2012; Ettinger et al., 2015; Cicero et al., 2019; Cheli, 2023). Individuals with high schizotypy showed similar cognitive function deficits as patients with schizophrenia (Ettinger et al., 2018; Bora, 2020), but with less severity (Rodríguez-Ferreiro et al., 2020). Research on schizotypy can explore the psychopathology and mechanisms of schizophrenia-spectrum disorders (Lenzenweger, 2015; Wang et al., 2022), making early intervention and prevention feasible (Kwapil and Barrantes-Vidal, 2015; Lenzenweger, 2018).

Despite a large number of studies on schizophrenia-spectrum disorders and P300 (Chen K. et al., 2014; Qiu et al., 2014), there are still some unresolved issues. First, previous studies rarely included non-clinical individuals with high schizotypal traits. A few studies discussed P300 in individuals at clinical high risk (CHR) and prodromal stage of schizophrenia (Graber et al., 2019; Hamilton et al., 2019; Higuchi et al., 2021). One small-sized study discussed P300 in individuals with high schizotypy below the diagnostic threshold of schizophrenia-spectrum disorders (Klein et al., 1999). Studying P300 characteristics among non-clinical individuals with high schizotypy can uncover the probable deficient auditory cognitive function related to non-clinical schizophrenia-like symptoms.

The second issue is the limited types of auditory stimuli used in the P300 paradigm. P300 is a long-lasting component generated in the temporal and parietal regions. It is elicited by an “oddball” paradigm consisting of frequent standard stimuli and infrequent target stimuli (Tsolaki et al., 2015; Habelt et al., 2020; Zeng et al., 2022). Most of the previous studies adopted deviant frequency as the target stimuli (Turetsky et al., 2015; Hamilton et al., 2019); however, the variant duration, an effective oddball, also induced the auditory P300 (Schenkein, 2006; St. George and Cone, 2020). Only one study used variant duration as an oddball for inducing P300 in patients with schizophrenia (Dzafic et al., 2021), it reported the reduced amplitude of duration P300 in patients with schizophrenia-spectrum disorders. In fact, psychoacoustic studies have proved the separation of frequency and duration in the primary auditory process (Levitin, 2006; Koelsch, 2012). Therefore, it is reasonable to assume that there are differences between the associations of schizophrenia-spectrum symptoms with frequency P300 and duration P300.

The third issue is about the parameters of target sounds in the oddball paradigm inducing P300, which varied greatly across studies on schizophrenia-spectrum. A meta-analysis revealed the decreased amplitude and increased latency of P300 as the frequency difference increases between target and standard stimuli (Jeon and Polich, 2003). It indicated that P300 measures can be dramatically influenced by auditory parameter when the schizophrenia group and control group are compared. However, the acoustic difference between frequent and infrequent stimuli in the oddball paradigm used in previous studies ranged from 50 Hz to 1,000 Hz (Mannan et al., 2001; Jeon and Polich, 2003; Graber et al., 2019; Higuchi et al., 2021). Therefore, using oddballs with distinct levels of difference between infrequent and frequent stimuli in a single study may elucidate the possible inconsistent relationship between schizophrenia-spectrum symptoms and P300 under different oddball conditions.

Considering these issues, this study aimed to investigate the frequency and duration P300 effect in non-clinical individuals with high schizotypy and explore the correlations between schizotypal traits and P300 under different auditory parameter conditions. Based on previous studies, the deficient P300 among CHR individuals and the first-degree biological relatives of patients with schizophrenia (Graber et al., 2019; Hamilton et al., 2019; Higuchi et al., 2021), non-clinical participants with high schizotypy were expected to show similar P300 impairment. Due to the separation of frequency and duration process (Levitin, 2006; Koelsch, 2012) and the modulatory effect of auditory parameters on P300 (Jeon and Polich, 2003), we predicted diverse patterns of correlation between schizotypal dimensions and P300 under different conditions.

## 2. Materials and methods

### 2.1. Participants

The subjects were screened similarly to our previous study (Deng et al., 2022). The schizotypal personality questionnaire (SPQ) was used to screen participants among 1,519 university students. The ordinal α coefficient of the Chinese-version SPQ is ranging from 0.74 to 0.84 (Fonseca-Pedrero et al., 2018). Totally, 31 participants with high schizotypal traits were chosen as the schizotypy group (SP), and 32 closely matched participants were chosen as the control group (CG). To be specific, after the preliminary SPQ test, an online survey was sent to individuals who reached in the top 15% of the total score. The survey included the SPQ retest and questions about basic demographic information. The following criteria were used during SP grouping: (1) Total SPQ score among the top 15% in two tests. (2) Exclusion of musicians, because there is evidence of a large P300 effect in musicians (Rabelo et al., 2015). (3) Absence of hearing impairment or substance abuse. (4) Absence of schizophrenia-spectrum disorders (in the individual or any of their first-degree relatives).

Participants in the CG were screened similarly to those in the SP. Detailedly, a survey was sent to the individuals who scored below the average in the preliminary SPQ test. According to the SP participants’ information, we searched for matching participants among candidates who met the predefined criteria (consistent with the SP except for below-average SPQ scores in both the preliminary test and retest) and sent them invitations to participate. Among the candidates who responded, we chose the most closely matched 32 participants and screened them for the CG. None of the SP or CG participants had head trauma or any neurological disorders, and all participants were right-handed. The basic information and SPQ scores of participants are listed in Table 1.

**TABLE 1:** Demographic characteristics of the study groups.

	Schizotypy			Control			*t/*χ^2^	*p*
	*n*	Mean/ percentage	*SD*	*n*	Mean/ percentage	*SD*		
Age (years)	31	20.36	2.30	32	20.52	2.45	-0.27	0.79
Gender							0.01	0.92
Male	12	38.7%		12	37.5%			
Female	19	61.3%		20	62.5%			
Parental SES	31	-0.01	0.26	32	0.01	0.32	-0.35	0.73
Education	31			32			0.13	0.72
Undergraduates	28	90.3%		28	87.5%			
Postgraduates	3	9.7%		4	12.5%			
IQ	31	107.23	9.32	32	109.72	7.58	-1.17	0.25
Music background	31	2.77	0.72	32	2.69	0.90	0.42	0.67
SPQ total score	31	51.42	6.06	32	11.22	5.12	28.49	<**0**.**001**
Positive	31	22.23	3.55	32	6.17	2.93	19.63	<**0**.**001**
Negative	31	16.61	3.01	32	3.09	2.12	20.70	<**0**.**001**
Disorganized	31	12.58	1.90	32	1.95	1.63	23.85	<**0**.**001**
Anxiety	31	45.81	8.96	32	35.06	6.01	5.60	<**0**.**001**
Depression	31	7.32	6.07	32	1.34	1.43	5.42	<**0**.**001**

IQ, intelligence quotient; SD, standard deviation; SES, socioeconomic status; SPQ, schizotypal personality questionnaire, which contained three dimensions: ① Positive (perceptual-cognitive facet), ② Negative (interpersonal facet), ③ Disorganized.

The bold value means the between-group difference is significant.

The depression status was assessed with the Beck depression inventory (BDI; Beck and Beamesderfer, 1974), and anxiety was evaluated by a self-rating anxiety scale (SAS; Zung, 1971). The intelligence quotient (IQ) was estimated using the Chinese version of the brief Wechsler adult intelligence scale (edited by Hunan Medical College; Gong, 1992). The parental socioeconomic status (SES) comprised three dimensions, calculated using the formula (β1 * Z parental education + β2 * Z parental occupation + β3 * Z family property)/εf, which was developed by Chen Y. H. et al. (2014). The music background was quantified with Grison’s revised criteria (Grison, 1972; Abe et al., 2017). Because of the significant differences in depression and anxiety between groups, these scores were considered as covariates during statistical analysis.

The study was approved by the local Ethics Committee of the School of Psychology, Fujian Normal University, and was performed in full compliance with the Declaration of Helsinki. All participants provided written informed consent before data acquisition and were financially reimbursed.

### 2.2. ERP P300 recording

#### 2.2.1. Paradigm

An auditory oddball paradigm consisting of two blocks was used. Block 1 contained 800 sounds: 656 frequent non-target stimuli (82%, 1,000 Hz, 50 ms) and 144 infrequent target stimuli (18%). The target stimuli included two levels of frequency: 72 stimuli were 1,200 Hz (9%; large-variant), and 72 stimuli were 1,050 Hz (9%; small-variant).

Block 2 also contained 800 sounds: 656 frequent non-target stimuli (82%, 1,000 Hz, 50 ms) and 144 target stimuli (18%). The target stimuli included two levels of duration: 72 stimuli were 150 ms (9%; large-variant), and 72 stimuli were 100 ms (9%; small-variant).

Participants were seated with their eyes opened in a slightly reclined chair, and had to press a button once the target sound was heard. All the sounds were presented binaurally through over-ear headphones (Bose QC35II) in a pseudo-randomized order. The stimulus onset asynchrony (SOA) was 1,000 ms. The volume of all stimuli was consistent across participants, with a 10-ms rise/fall period. All participants were trained to ensure that they could discriminate between target and non-target tones. Participants were instructed to rest between blocks, and the presentation order of two blocks was balanced between groups with the ABBA method.

#### 2.2.2. Recording

Recording took place in an anechoic and electrically shielded chamber. Electroencephalographic (EEG) data were acquired at a sampling rate of 1,000 Hz using a 64-channel NeuroScan EEG system (International 10–20 layout; Neuroscan, Germany). Bipolar recordings of horizontal and vertical electro-oculogram activity were obtained from the supra-/sub-orbital and lateral canthus sites, respectively. The impedances of all electrodes were monitored for each subject to verify that its value was under 10 kΩ. The electrical activity was recorded with an analog bandpass filter of 0.1–100 Hz, and with average reference.

Data were analyzed using EEGLAB (v13.6.5b). Filters were applied from 0.1–30 Hz, with a notch filter at 50 Hz. The epoch was 900 ms, including a 100 ms pre-stimulus baseline. Eye-blink and eye movement artifacts were corrected through independent component analysis (ICA). Epochs containing artifacts (exceeding ± 75 μV) at each electrode were excluded from the analysis. Data from 3 participants in CG and 2 participants in SP were excluded because more than one-third of their epochs had artifacts.

### 2.3. Statistical analysis

According to previous studies on P300 (Tsolaki et al., 2015; Habelt et al., 2020; Kim et al., 2020; Higuchi et al., 2021; Zeng et al., 2022), we defined 300–500 ms after the target sound as time-window, and a positive maximum peak amplitude was detected within this time window by ERPLAB (v7.0.0). Peak latency was defined as the time point of maximum positive amplitude within the specific latency window. ERP waveforms were acquired by across-trial averaging of PZ and CPZ electrode sites. Data for the frequency P300 (fP300) and duration P300 (dP300) blocks were processed separately as they were measured separately. The raw data is available in the Supplementary Material.

Statistical analyses were performed using SPSS (IBM, USA). Independent sample *t*-test or chi-square test were used to compare the demographics between groups. Amplitude and latency of fP300 and dP300 were analyzed using repeated measures analysis of covariance (ANCOVA). The oddball conditions (large-variant target and small-variant target) were considered as the within-subject factors and groups (SP and CG) were considered as the between-subject factors. Depression and anxiety scores were considered as covariates to eliminate the latent impact of worse mood in combination with high schizotypal traits. Bonferroni correction was used for multiple tests. Partial correlation was used to explore the associations between P300 and SPQ scores, with the depression and anxiety scores as covariates.

## 3. Results

### 3.1. Demographic comparisons

As presented in Table 1, schizotypy, anxiety, and depression scores were significantly higher in the SP than in the CG (*p* < 0.001). There were no statistical differences in age, gender, parental SES, education, IQ, and musical background between the two groups.

### 3.2. P300 effect under different oddball conditions in two groups

**FIGURE 1:**
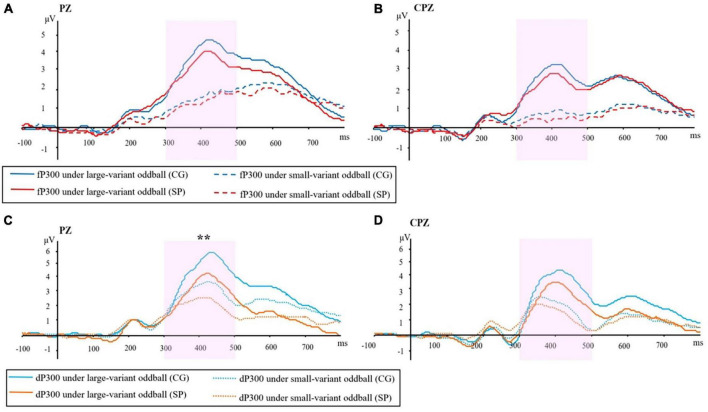
ERP waveforms in two groups under different conditions of oddball: **(A)** Frequency P300 at the PZ site. **(B)** Frequency P300 at the CPZ site. **(C)** Duration P300 at PZ site. **(D)** Duration P300 at CPZ site. P300 amplitude was evaluated as the maximum between 300–500 ms. fP300, frequency P300; dP300, duration P300; CG, control group; SP, schizotypy group; μV, microvolt; ms, millisecond. **p < 0.01.

**FIGURE 2:**
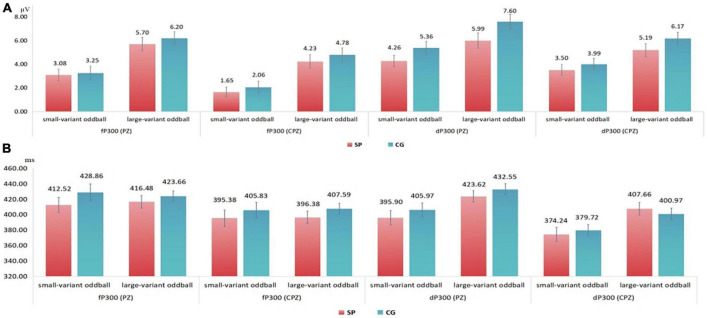
**(A)** Means and standard errors of the detected peak amplitude in 300–500 ms time window. **(B)** Means and standard errors of the detected latency in 300–500 ms time window. fP300, frequency P300; dP300, duration P300; CG, control group; SP, schizotypy group; μV, microvolt; ms, millisecond.

For the fP300 amplitude at PZ site (Figure 1A), the main effect of group [*F*(1,54) = 1.15, *p* = 0.288, η_*p*_^2^ = 0.021] and interactive effect [*F*(1,54) = 0.41, *p* = 0.52, η_*p*_^2^ = 0.008] were not significant. A main effect of oddball variant-level condition was found [*F*(1,54) = 11.01, *p* = 0.002, η_*p*_^2^ = 0.169]. The means and standard errors of P300 amplitude are shown in Figure 2A. For the fP300 latency at the PZ site, the main effect of group [*F*(1,54) = 3.29, *p* = 0.075, η_*p*_^2^ = 0.057], interaction [*F*(1,54) = 0.051, *p* = 0.823, η_*p*_^2^ = 0.001], and the main effect of oddball condition [*F*(1,54) = 0.082, *p* = 0.775, η_*p*_^2^ = 0.002] were not significant. The means and standard errors of P300 latency are shown in Figure 2B.

**FIGURE 3:**
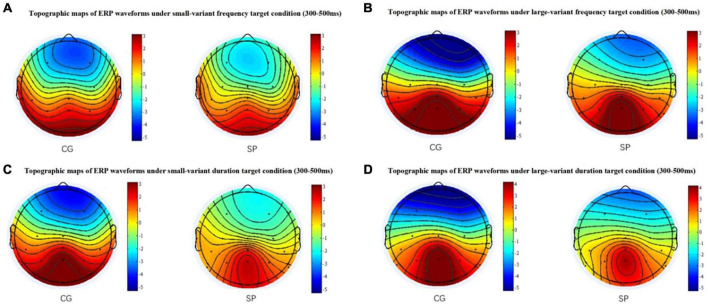
The topographic maps between 300 ms and 500 ms. **(A)** The topographic maps for the small-variant frequency oddball condition. **(B)** The topographic maps for the large-variant frequency oddball condition. **(C)** The topographic maps for the small-variant duration oddball condition. **(D)** The topographic maps for the large-variant duration oddball condition. CG, control group; SP, schizotypy group.

For the fP300 amplitude at the CPZ site (Figure 1B), there was neither a significant main effect of group [*F*(1,54) = 0.434, *p* = 0.513, η_*p*_^2^ = 0.008], nor a significant interactive effect [*F*(1,54) = 0.407, *p* = 0.526, η_*p*_^2^ = 0.007], but the main effect of oddball variant condition was significant [*F*(1,54) = 6.601, *p* = 0.013, η_*p*_^2^ = 0.109]. Similar to the results at the PZ site, there was not any significant main or interactive effect (*p* > 0.05) for the latency of fP300 at the CPZ site. In addition, combining the waveforms and topographic maps (Figure 3) of fP300 induced by small variant oddballs, although the ERP was positive deflection, the maximum effect was not in the conventionalized time window of P300 (300–500 ms), but around 600 ms.

As shown in Figure 1C, for the dP300 amplitude at the PZ site, there was a significant main effect of group [*F*(1,54) = 7.455, *p* = 0.009, η_*p*_^2^ = 0.121], that the amplitude of dP300 was decreased in the SP (Figure 2A). The effect of oddball variant condition [*F*(1,54) = 0.072, *p* = 0.790, η_*p*_^2^ = 0.001], and the interaction [*F*(1,54) = 2.917, *p* = 0.093, η_*p*_^2^ = 0.051] were not significant. For the dP300 latency at the PZ site, the main effect of oddball variant condition [*F*(1,54) = 2.429, *p* = 0.125, η_*p*_^2^ = 0.043], group [*F*(1,54) = 3.382, *p* = 0.071, η_*p*_^2^ = 0.059], and the interactive effect were not significant [*F*(1,54) = 0.971, *p* = 0.329, η_*p*_^2^ = 0.018].

For the dP300 amplitude at the CPZ site (Figure 1D), no significant effect was found for group [*F*(1,54) = 1.770, *p* = 0.189, η_*p*_^2^ = 0.032], oddball variant condition [*F*(1,54) = 0.069, *p* = 0.794, η_*p*_^2^ = 0.001], or interaction [*F*(1,54) = 3.636, *p* = 0.062, η_*p*_^2^ = 0.063]. Similar to the results at the PZ site, there was not any significant main or interactive effect (*p* > 0.05) for dP300 latency at CPZ site.

### 3.3. The relationship between P300 and schizotypal traits in two groups

**FIGURE 4:**
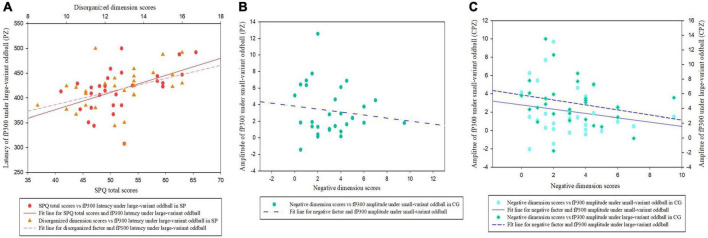
Scatter plots showing a linear correlation between the fP300 effect and SPQ (total and dimensional scores). **(A)** The relationships between fP300 latency at the PZ site and schizotypy in the SP. **(B)** The relationship between fP300 amplitudes at the PZ site and schizotypal negative facet in the CG. **(C)** The relationships between fP300 amplitudes at the CPZ site and schizotypal negative facet in the CG. fP300, frequency P300; CG, control group; SP, schizotypy group.

As shown in Figure 4A, in the SP, the latency of fP300 at the PZ site under large-variant oddball condition was significantly partial correlated with total SPQ scores (*r*_*p*_ = 0.451, *p* = 0.018), and disorganized dimension scores (*r*_*p*_ = 0.381, *p* = 0.050). It means the more disorganized symptoms, the longer latency of fP300 in the high schizotypy group. No significant association was found between schizotypal traits and fP300 at the PZ site under small-variant oddball condition (*p* > 0.05). In addition, no significant correlation was found between schizotypal traits and the fP300 at CPZ (*p* > 0.05) in the SP.

In the CG (Figure 4B), we found a significantly negative correlation between the negative dimension score of SPQ and fP300 amplitude at the PZ site under small-variant oddball condition (*r*_*p*_ = −0.393, *p* = 0.043). Furthermore, as shown in Figure 4C, the negative factor of schizotypy was inversely correlated with fP300 amplitude at the CPZ site under both small variant oddball condition (*r*_*p*_ = −0.406, *p* = 0.035) and large variant oddball condition (*r*_*p*_ = −0.408, *p* = 0.035). It means the more negative symptoms of schizotypy, the weaker amplitude of fP300 in the control group. No significant links were found between fP300 and the positive or disorganized dimension scores in the CG (*p* > 0.05).

There were not any significant correlations between SPQ and the amplitude or the latency (*p* > 0.05) of dP300 at both PZ and CPZ sites in the SP.

**FIGURE 5:**
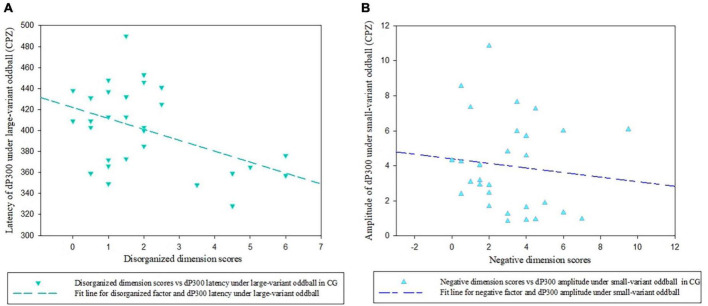
Scatter plots showing a linear correlation between the dP300 effect and SPQ dimensional scores. **(A)** The relationship between dP300 latency at the CPZ site and schizotypal disorganized facet in the CG. **(B)** The relationship between dP300 amplitudes at the CPZ site and schizotypal negative facet in the CG. dP300, duration P300; CG, control group.

However, In the CG, we found a significantly negative relationship between the disorganized dimension scores and dP300 latency at the CPZ site under large-variant oddball condition (*r*_*p*_ = −0.518, *p* = 0.006; Figure 5A), indicating the shorter latency with the more disorganized symptoms in participants from the CG. In addition, a significantly negative association was found between negative factor scores and dP300 amplitude under small-variant oddball condition (*r*_*p*_ = −0.410, *p* = 0.034; Figure 5B). No other significant correlations between dP300 and schizotypy were found in the CG (*p* > 0.05).

## 4. Discussion

As far as we know, this is the first study assessing the auditory P300 using both duration and frequency stimuli in the oddball paradigm among non-clinical individuals with high schizotypy. It is also the first study evaluating the P300 amplitude and latency under different variant parameter conditions in this sample.

The prior hypotheses of our study were verified partly. The P300 effect, particularly the dP300 amplitude, was markedly impaired in individuals with high schizotypal traits. In addition, diverse patterns of relationship were found between schizotypal dimensions and P300 effects under different oddball conditions. The current results have been discussed from two viewpoints in the following sections.

### 4.1. Schizotypy continuum and the P300 deficits

Although the significant between-group difference was shown in the duration P300 in our study, weaker P300 amplitude existed in all tests. These findings are similar to those from clinical individuals with schizophrenia-spectrum disorders (Wang et al., 2003; Bramon et al., 2004; Higuchi et al., 2013, 2021; Kim et al., 2015, 2020), and consistent with the previous non-clinical report (Klein et al., 1999), indicating schizophrenia-related impairment of attention and working memory in acoustic information process (Kim et al., 2015). This process impairment may be related to the poor information flow in individuals with high schizotypy (Hu et al., 2020), which was supported by the evidence of machine learning study (Zandbagleh et al., 2022a), that reduced connectivity between prefrontal and parietal regions in the beta band, and decreased frontal connectivity in the alpha band in participants with high schizotypy.

The current findings support the viewpoint that schizotypy is an “endophenotype” on the path to schizophrenia (Grant, 2015; Wang et al., 2022). The deficits of a top-down auditory process reflected by the weak P300 in our non-clinical sample with high schizotypy is in favor of the widely accepted theory of schizotypy-continuum (Ettinger et al., 2015; Cicero et al., 2019; Nesic et al., 2019; Cheli, 2023), which refers to a continuum of schizophrenia-like manifestations in the general population (Claridge and Beech, 1995), from healthy to clinical levels (Grant et al., 2018).

Most correlations found in the CG supported the between-group difference of P300 amplitude. The amplitudes of frequency and duration P300 were negatively associated with schizotypy, particularly with negative dimension. It means that weaker auditory P300 amplitude was associated with more symptoms of schizophrenia-like social withdrawal and flattened affect. These results are consistent with previous studies from individuals with clinical manifestations (Ford, 1999; Mathalon et al., 2000; Bruder et al., 2001; Perlman et al., 2015). However, no significant correlation was found between positive schizotypal dimension and P300 in our study, inconsistent with the previous result from patients with schizophrenia (O’Donnell et al., 1993). Therefore, based on our findings and those from previous studies (Bruder et al., 2001; Perlman et al., 2015; Kim et al., 2018), P300 may be more sensitive to the negative dimension of schizophrenia-spectrum regardless of diagnostic threshold.

Although non-clinical individuals with high schizotypy have a schizophrenia-like impaired cognitive function, it does not imply that the impairment is entirely and linearly correlated with schizophrenia-spectrum symptoms. According to the “Connectivity Decompensation Hypothesis” (Mohr and Claridge, 2015; Wang et al., 2022), individuals with high schizotypal traits exhibit both decompensatory and compensatory effects, which may prevent schizophrenia-spectrum symptom exacerbation. The inverse correlations between P300 latency and disorganized factor in the SP and CG (Figures 4A, 5A), could be explained by the decompensatory and compensatory effects. A positive association between frequency P300 latency and disorganized factor was found in the SP, which is consistent with previous results from clinical samples (Bramon et al., 2004; Higuchi et al., 2021). Whereas, we found that the latency of duration P300 was shortened with a mild increment of disorganized traits in the CG. Therefore, shorter latency of dP300 in CG possibly indicates faster temporal information processing for maintaining the normal velocity of voluntary attention to external sounds in non-clinical individuals. For the SP, the lack of inverse relationship between duration P300 latency and disorganized factors, but the presence of a link between frequency P300 latency and disorganized factors, may indicate an ineffective compensation (Mohr and Claridge, 2015; Wang et al., 2022).

### 4.2. Stimuli characteristics used in the study of P300 and schizophrenia-spectrum

Based on current findings (Figure 1), we assume that duration P300 may be more sensitive than frequency P300, when comparing the non-clinical samples with high and low schizotypal traits. Although most of the previous studies of P300 in schizophrenia-spectrum disorders adopted deviant frequency stimuli as the target in oddball paradigms (Turetsky et al., 2015; Hamilton et al., 2019), deviant duration stimuli are anticipated to be increasingly used in future studies of auditory P300 in schizophrenia-spectrum.

From a psychoacoustics perspective, the low amplitude of duration P300 reflects the impairment of auditory temporal information processing (Schenkein, 2006; Gibbons, 2022). It is reminiscent of a previous study revealed a marked deficiency in discrimination and prediction of temporal information in individuals with schizophrenia (Turgeon et al., 2012). In fact, auditory temporal information processing is highly and socially meaningful (Wang, 2015). It involves rhythm pattern construction, meter extraction, and tempo perception based on speech and non-speech sound cues (Patel, 2010), which are indispensable for synchronicity of cooperative activity during human socialization and evolution (Wallin et al., 2001; Levitin, 2006; Koelsch, 2012). Therefore, hypoactive working memory for duration information in SP may warrant negative facets of schizophrenia-spectrum, such as interpersonal detachment, low gregariousness, and social withdrawal (Owen et al., 2016; Cheli, 2023), and needs to be explored further.

Although the interactive effects were not statistically significant, the correlation analyses revealed diverse patterns of relationship between schizotypal traits and P300 under different oddball conditions. Specifically, the association between negative schizotypy and P300 effect under small variant oddball condition manifested in the amplitude index. While the association between disorganized schizotypy and P300 effect under large variant oddball condition manifested in the latency index. In addition, attention must be paid to the complicated relationship between disorganized factors and P300 latency, that the trend may be inverse with the target stimuli characteristics alter and the symptoms severity vary.

At present, P300 has been studied as a potential neural biomarker to achieve the goal of improving the accuracy in detection of early episodes of psychosis (Zandbagleh et al., 2022b), or as a predictor of treatment and prognosis (Kim et al., 2020, 2015), that are of significance in the research on psychopathology and early-intervention of schizophrenia-spectrum. Considering the various associated tendencies between auditory P300 effect and schizotypy dimensions in our result, elaborated characteristics and parameters should be used in the oddball paradigm to enhance the application of P300 in future studies of schizophrenia-spectrum.

### 4.3. Limitations

This study had several limitations. On the one hand, although the duration and frequency of targets were considered in the oddball paradigm, additional characteristics of psychoacoustic stimuli such as intensity and location should be considered in future studies of P300 among schizophrenia-spectrum individuals to improve the predictability and application of auditory P300. On the other hand, we used extreme-group design in the current study because previous studies demonstrated that extreme-group design may be more suitable for schizotypy studies than those with non-extreme-group design (Bora, 2020). However, the relationship between auditory P300 and schizotypy should be examined using a non-extreme group design in the future to verify the generalizability of the schizotypy-continuum theory.

## 5. Conclusion

In summary, this study found that the amplitude of duration P300 was markedly impaired in non-clinical individuals with high schizotypal traits, indicating schizophrenia-related deficiency of attention and working memory in the acoustic information process in non-clinical individuals and supporting schizotypy-continuum theory. In addition, we discovered diverse relationship patterns between schizotypal dimensions and P300 effects under different oddball parameter conditions. The association between the negative schizotypy and P300 under the small variant oddball condition was manifested in the amplitude index, while the association between the disorganized schizotypy and P300 under the large variant oddball condition was manifested in the latency index. These findings indicate that characteristics and parameters of target stimuli must be considered when using an auditory oddball paradigm among individuals with schizophrenia-spectrum symptoms.

## Data availability statement

The original contributions presented in this study are included in the article/Supplementary material, further inquiries can be directed to the corresponding author.

## Ethics statement

The studies involving human participants were reviewed and approved by the Ethics Committee of the School of Psychology, Fujian Normal University. The patients/participants provided their written informed consent to participate in this study.

## Author contributions

JD, ZL, and YY: conceptualization. JD, SC, and YZ: data collection and curation. JD: formal analysis and writing—original draft. YO and YS: funding acquisition. JD, YO, and YS: methodology. YY and YO: supervision. JD and YY: writing—review and editing. All authors contributed to the article and approved the submitted version.
